# Data on renal cortical elasticity using dynamic shear wave elastography among healthy population

**DOI:** 10.6026/973206300214896

**Published:** 2025-12-15

**Authors:** Manisha Oraon, Suresh Kumar Toppo, Rajeev Kumar Ranjan, Anima Ranjni Xalxo, Nisha Rai, Riya Agarwal, Sonali Priyadarsini Reddy, Mohd Ismail

**Affiliations:** 1 Department of Radio-diagnosis, Rajendra Institute of Medical Sciences, Ranchi, Jharkhand, India

**Keywords:** Renal cortical elasticity, shear wave elastography, chronic kidney disease, normative data, ElastPQ, kidney stiffness

## Abstract

Chronic kidney disease (CKD) requires early diagnostic tools beyond conventional methods. Therefore, it is of interest to establish normative renal cortical elasticity values using point Shear Wave Elastography (pSWE) in 289 healthy adults aged 20-50 years in Ranchi, India. Mean elasticity was 1.53 ± 0.196 m/sec (right kidney) and 1.52 ± 0.130 m/sec (left), with no significant side difference. Elasticity showed no association with age, gender, renal dimensions, or cortical thickness, but demonstrated a significant negative correlation with BMI and parenchymal thickness. These reference values support future SWE-based evaluation of renal pathologies in the Indian population.

## Background:

Chronic kidney disease (CKD) affects approximately 10% of the global population and represents a progressive condition leading to end-stage renal disease (ESRD) [1]. The kidneys play a crucial role in maintaining homeostasis through filtration of approximately 200 liters of fluid daily, regulating electrolyte balance, acid-base equilibrium, and blood pressure while producing essential hormones [2]. Renal fibrosis serves as a critical contributor to CKD progression, encompassing pathological changes including interstitial fibrosis, glomerulosclerosis, tubular atrophy, and vascular alterations [3]. Traditional diagnostic methods for CKD evaluation include blood tests such as serum urea, creatinine, and urinary protein measurements, which track disease progression but demonstrate limited sensitivity, particularly in early stages [4]. While kidney biopsy remains the definitive option for assessing fibrosis, it presents an invasive procedure with multiple contraindications including coagulopathy disorders, polycystic kidney disease, urinary tract obstruction, and upper urinary tract infections [5]. Additionally, biopsy carries risks of bleeding complications, with reported rates ranging from 1-7% depending on patient factors and operator expertise [6]. Renal imaging techniques, including ultrasonography (USG), computed tomography (CT), and magnetic resonance imaging (MRI), offer non-invasive alternatives for evaluating kidney structure, function, and blood flow [7]. Studies have demonstrated correlations between renal fibrosis and parameters such as pole-to-pole length, parenchymal thickness, and resistive index [8]. However, conventional ultrasound parameters lack the sensitivity and specificity required for accurate early-stage CKD evaluation, as morphological alterations often remain undetectable until significant parenchymal damage has occurred [9]. Ultrasound elastography has emerged as a promising non-invasive technique for quantitatively assessing tissue elasticity through various methods [10]. This technology has gained widespread acceptance in liver fibrosis assessment, with established protocols and reference values [11].

Shear wave elastography (SWE), specifically, enables non-invasive measurement of tissue stiffness by tracking shear wave propagation through tissues, with stiffer tissues facilitating faster wave propagation [12]. The Young's modulus (E) can be calculated using the formula E = 3ρcs^2^, where ρ represents tissue density and cs denotes shear wave speed, providing an objective measurement of tissue hardness [13, 14- 15]. However, significant variability exists across studies due to differences in equipment, measurement techniques and population characteristics [16]. Despite these advances, substantial gaps remain in the literature regarding normative renal elastography data, particularly for Asian populations. Most existing studies focus on Western populations or patients with established renal pathologies [17]. Furthermore, the influence of demographic and anthropometric factors on renal elasticity measurements remains incompletely understood, with conflicting reports regarding the effects of age, gender, and body mass index [18, 19]. Therefore, it is of interest to establish comprehensive normative data for renal cortical elasticity using dynamic shear wave elastography in a healthy Indian population and evaluate the influence of various demographic, anthropometric, and renal structural factors on these measurements.

## Materials and Methods:

## Study design and setting:

This facility-based observational cross-sectional study was conducted at the Department of Radiodiagnosis, Rajendra Institute of Medical Sciences (RIMS), Ranchi, Jharkhand, India. The study duration was 18 months, from June 2023 to January 2025, comprising 3 months for literature review, 12 months for data collection and analysis, and 3 months for report writing and thesis submission.

## Sample size calculation:

The sample size was calculated using the formula n = 4 x SD^2^/d^2^, where SD represents the standard deviation and d denotes the desired precision. Based on previous research [14], the average standard deviation of kidney lengths was 0.85 cm. With a desired precision (d) of 10%, the calculated sample size was 289 participants.

## Study population:

The study population comprised 289 individuals including outpatients, inpatients, and their attenders at RIMS, Ranchi. Participants were selected based on predefined eligibility criteria

## Inclusion criteria:

[1] Age between 20-50 years

[2] Normal kidney function (serum urea ≤ 40 mg/dL, creatinine ≤ 1.3 mg/dL)

[3] No abnormal findings on grayscale ultrasonography

[4] Willingness to participate and provide written informed consent

## Exclusion criteria:

[1] Unwillingness to participate

[2] Deranged kidney function (serum urea > 40 mg/dL, creatinine > 1.3 mg/dL)

[3] Abnormal ultrasound findings (renal cysts, nephrolithiasis, hydronephrosis, ureterolithiasis, bladder calculi, ascites)

[4] Known history of diabetes, hypertension, or trauma

[5] Inability to comply with breath-holding instructions during examination

## Ethical considerations:

Ethical clearance was obtained from the Institutional Ethics Committee of RIMS, Ranchi (Reference No: RIMS/IEC/2023/045). The study adhered to the tenets of the Declaration of Helsinki, and written informed consent was obtained from all participants before their inclusion. Participants were assured of confidentiality and their right to withdraw from the study at any time without penalty.

## Data collection:

Demographic data including age, sex, weight, and height were recorded for all participants. Body mass index (BMI) was calculated and categorized according to World Health Organization guidelines for Asian populations: underweight (<18.5 kg/m^2^), normal weight (18.5-22.9 kg/m^2^), overweight (23.0-27.4 kg/m^2^), and obese (≥27.5 kg/m^2^).

## Ultrasound examination and elastography:

All participants underwent a transabdominal ultrasound examination and shear wave ultrasound elastography using a PHILIPS Affiniti 70G machine with a convex probe (C5-1, 1-5 MHz). Before examination, participants were pre-screened for residual urine in the supine position. All measurements were performed by a single experienced radiologist to ensure consistency.

Participants were positioned in lateral decubitus after emptying the urinary bladder.

The following renal parameters were measured:

[1] Kidney length: Distance from superior to inferior pole in the coronal plane

[2] Kidney width: Distance from renal hilum to renal capsule at the mid-pole in the coronal plane

[3] Parenchymal thickness: Distance from outer margin of renal sinus to renal capsule at the mid-pole in the coronal plane

[4] Cortical thickness: Distance from renal capsule to base of medullary pyramid at the mid-pole

[5] Skin-to-cortex distance: Distance from skin to outer margin of renal cortex at the mid-pole

For elastography measurements, point Shear Wave Elastography (pSWE) was performed using ELAST PQ software based on Acoustic Radiation Force Impulse (ARFI) technology. The probe was placed steadily with minimal compression, and participants were instructed to hold their breath in full inspiration for a few seconds to minimize respiratory motion artifacts. A region of interest (ROI) box of 1.0 x 0.5 cm (predefined by the manufacturer) was positioned in the renal cortex at the middle third of the kidney, excluding the medulla as much as possible. The main axis of the ROI box was aligned parallel to the main axis of the medullary pyramids at the mid-pole. Five valid measurements of shear wave velocity were taken for each kidney, and the average value was recorded in meters per second (m/s).

## Statistical analysis:

Data were analyzed using Statistical Package for Social Sciences (SPSS) version 26.0 software. Descriptive statistics were presented as mean ± standard deviation (SD) for continuous variables and frequencies (percentages) for categorical variables. For multivariate analysis, one-way ANOVA test was used. Unpaired sample t-test was employed to compare means between independent groups. Pearson's correlation coefficient was used to assess relationships between continuous variables. Multiple regression analysis with the enter method was performed to identify factors influencing elasticity values. A p-value < 0.05 was considered statistically significant.

## Results:

The study included 289 participants with a mean age of 38.35 ± 8.43 years (range: 20-50 years). The majority of participants were male (77.2%, n = 223), with females comprising 22.8% (n = 66). The mean BMI was 24.18 ± 3.46 kg/m^2^, with most participants classified as overweight (42.6%, n = 123) or normal weight (37.0%, n = 107). Renal function parameters were within normal limits, with mean serum urea of 23.43 ± 4.64 mg/dL and mean serum creatinine of 0.79 ± 0.13 mg/dL. Detailed demographic and clinical characteristics are presented in Table 1 (see PDF). The mean kidney length was 9.46 ± 1.00 cm for the right kidney and 9.68 ± 0.72 cm for the left kidney, with a statistically significant difference between them (p = 0.002). Similarly, the left kidney demonstrated significantly greater width (5.08 ± 2.59 cm vs. 4.59 ± 0.66 cm, p = 0.002), parenchymal thickness (1.90 ± 0.29 cm vs. 1.78 ± 0.30 cm, p < 0.001), and cortical thickness (0.94 ± 0.19 cm vs. 0.88 ± 0.16 cm, p < 0.001) compared to the right kidney. The skin-to-cortex distance was slightly greater for the right kidney (4.55 ± 0.93 cm vs. 4.37 ± 0.97 cm, p = 0.032). The mean elasticity values were 1.53 ± 0.196 m/sec for the right kidney and 1.52 ± 0.130 m/sec for the left kidney, with no statistically significant difference between them (p = 0.056). The overall average elasticity value for both kidneys was 1.52 ± 0.13 m/sec. Kidney dimensions and elasticity values are summarized in Table 2 (see PDF). Pearson's correlation analysis revealed no significant correlation between average elasticity values and age (r = 0.005, p = 0.931) or gender (p = 0.816). Similarly, kidney dimensions including length (r = 0.035, p = 0.558), width (r = -0.035, p = 0.559), and cortical thickness (r = 0.040, p = 0.499) showed no significant correlation with elasticity values. However, a significant negative correlation was observed between average elasticity values and BMI (r = -0.207, p < 0.001) and parenchymal thickness (r = -0.163, p = 0.005). The skin-to-cortex distance did not demonstrate a significant correlation with elasticity values (r = 0.014, p = 0.814). The correlation analysis results are presented in Table 3 (see PDF). Multiple regression analysis confirmed that BMI (β = -0.232, p < 0.001) and parenchymal thickness (β = -0.222, p = 0.001) were significant negative predictors of elasticity values, while age, gender, kidney length, width, cortical thickness, and skin-to-cortex distance did not show significant predictive value.

## Discussion:

This study established normative values for renal cortical elasticity in a healthy adult population, providing important reference data for both clinical and research applications. The mean elasticity values for the right and left kidneys were very similar, indicating comparable tissue stiffness and functional integrity between the two kidneys [14, 20, 21 and 22]. These values were consistent with previous studies in similar populations, but they were lower than those reported in several Western cohorts, likely due to differences in equipment, measurement techniques, population characteristics, and genetic factors. No significant associations were observed between renal elasticity and age in this study, which aligns with some previous reports [23, 24]. However, other studies have reported a negative correlation between age and elasticity values [25], suggesting that age-related changes might become more apparent in older populations that were not included in the current study. Similarly, no significant influence of gender on renal elasticity was observed, consistent with prior findings [26], although some studies have reported higher elasticity values in females [15]. The uneven gender distribution in the current cohort may have limited the ability to detect gender-related differences. A significant negative correlation between BMI and renal elasticity was observed [19], suggesting that individuals with higher BMI tend to have lower elasticity values. This may be due to increased perirenal fat affecting shear wave propagation or metabolic factors associated with obesity that alter tissue properties. Parenchymal thickness also showed a negative correlation with elasticity values, which may reflect complex interactions between renal structure and tissue mechanics. In contrast, kidney length, width, and cortical thickness were not significantly correlated with elasticity, highlighting those conventional ultrasound measurements alone may not reliably predict renal tissue stiffness. The establishment of normative renal elasticity data has important clinical implications. These reference values provide a baseline for detecting early renal damage in pathological conditions such as diabetic nephropathy, hypertensive nephrosclerosis, and glomerulonephritis. Shear wave elastography has shown high diagnostic accuracy in assessing renal fibrosis [27, 28], but challenges remain in standardizing measurement techniques. Factors such as tissue anisotropy, vascularity, urinary tract pressure, and respiratory motion can influence results [29, 30] and must be considered in clinical applications. Future research should include longitudinal studies to investigate the relationship between changes in renal elasticity and disease progression. Multicenter studies with larger and more diverse populations are also necessary to validate findings and establish population-specific reference values. This would improve the clinical utility of renal elastography in detecting early renal pathologies and monitoring treatment outcomes.

## Conclusion:

Data on renal cortical elasticity using dynamic shear wave elastography among healthy Indian population is needed. The mean elasticity values of 1.53 ± 0.196 m/sec for the right kidney and 1.52 ± 0.130 m/sec for the left kidney provide essential reference values for future clinical applications and research. We identified significant negative correlations between elasticity values and BMI and parenchymal thickness, while no significant associations were found with age, gender, kidney length, width, or cortical thickness. These findings lay a crucial foundation for the clinical application of renal elastography in the early detection and monitoring of renal pathologies.
